# Identification of Differentially Expressed Genes Related to Floral Bud Differentiation and Flowering Time in Three Populations of *Lycoris radiata*

**DOI:** 10.3390/ijms232214036

**Published:** 2022-11-14

**Authors:** Guanghao Cheng, Fengjiao Zhang, Xiaochun Shu, Ning Wang, Tao Wang, Weibing Zhuang, Zhong Wang

**Affiliations:** 1 Institute of Botany, Jiangsu Province and Chinese Academy of Sciences (Nanjing Botanical Garden Mem. Sun Yat-Sen), Nanjing 210014, China; 2 Jiangsu Key Laboratory for the Research and Utilization of Plant Resources, Jiangsu Provincial Platform for Conservation and Utilization of Agricultural Germplasm, Nanjing 210014, China

**Keywords:** *Lycoris radiata*, floral bud differentiation, flowering time, paraffin section, transcriptome, WGCNA

## Abstract

The transition from vegetative to reproductive growth is important for controlling the flowering of *Lycoris radiata*. However, the genetic control of this complex developmental process remains unclear. In this study, 18 shoot apical meristem (SAM) samples were collected from early-, mid- and late-flowering populations during floral bud differentiation. The histological analysis of paraffin sections showed that the floral bud differentiation could be divided into six stages; the differentiation time of the early group was earlier than that of the middle and late groups, and the late group was the latest. In different populations, some important differential genes affecting the flowering time were identified by transcriptome profiles of floral bud differentiation samples. Weighted gene co-expression network analysis (WGCNA) was performed to enrich the gene co-expression modules of diverse flowering time populations (FT) and floral bud differentiation stages (ST). In the MEyellow module, five core hub genes were identified, including *CO14*, *GI*, *SPL8*, *SPL9*, and *SPL15*. The correlation network of hub genes showed that they interact with SPLs, AP2, hormone response factors (auxin, gibberellin, ethylene, and abscisic acid), and several transcription factors (MADS-box transcription factor, bHLH, MYB, and NAC3). It suggests the important role of these genes and the complex molecular mechanism of floral bud differentiation and flowering time in *L. radiata*. These results can preliminarily explain the molecular mechanism of floral bud differentiation and provide new candidate genes for the flowering regulation of *Lycoris*.

## 1. Introduction

Flowering is an important process in the life history of higher plants, which suffer the developmental transition from the nutritional stage to the reproductive stage of the shoot apical meristem [1]. The flowering time not only has an impact on ecological and evolutionary processes but also affects the success of plant reproduction, which is crucial for plant breeding. Therefore, it is important to explore the genetic mechanisms of flowering, especially for crops and horticultural species [2]. Studies have shown that plant flowering is mainly determined by a combination of environmental factors, such as temperature, light, and endogenous factors (plant age and plant hormones) [3]. Environmental and endogenous factors contribute to the regulation by five major flowering pathways, including the photoperiod pathway, the vernalization pathway, the age pathway, the gibberellin pathway, and the autonomous pathway [4]. Under the combination of these pathways, an integrated regulatory network crosstalks each other and regulates floral transformation through guidance signals, such as *FLOWERING LOCUS T* (*FT*), *CONSTANS* (*CO*), *SUPPRESSOR OF CONSTANS1* (*SOC1*), *LEAFY* (*LFY*), *APETALA1* (*AP1*), *APETALA2* (*AP2*), and *FLOWERING LOCUS C* (*FLC*) [5].

The flowering mechanism of the model plant *Arabidopsis thaliana* has been well studied, and a clear regulatory network has been described [6,7,8]. Nevertheless, new regulatory genes and mechanisms are still being continually reported [9,10,11], indicating that the regulatory network of flowering is not only complex but also highly variable in different species. For ornamental flowers, the flower is the most important trait, and many studies have focused on the flowering pathways and gene functions. For example, the APETALA2/ethylene-responsive element-binding factors (AP2/ERFs) are a large group of factors that are mainly found in plants, serving as an important regulator in plant flowering [12]. *AfAP2-2*, a gene that encodes an AP2 homolog, was identified in the genome of *Aechmea fasciata*, a popular ornamental flowering bromeliad. In *AfAP2-2*-overexpressing (*AfAP2-2-OX*) Arabidopsis, the bolting time exhibited no significant difference from that of the wild-type (WT) under long-day (LD) conditions, but flowering was delayed in *AfAP2-2-OX* Arabidopsis in short-day (SD) conditions [13]. In chrysanthemum, ethylene-responsive element-binding protein (ERF) transcription factors were involved in regulating photoperiodic flowering, and overexpressing *CmERF110* led to earlier flowering [14]. *AGL* (*AGAMOUS-like*) and *SEP3* (*SEPALLATA3-like*) are important floral meristem identity genes. Adal et al. (2021) found that the expression of full-length cDNAs encoding lavender LaAG-like and LaSEP3-like transcription factors could induce early flowering and impact leaf morphology at a strong expression level in Arabidopsis [15]. *SVP* plays an important role in gibberellin and autonomous pathways. The coding sequence of the *SVP* ortholog is referred to as *SVL*. The overexpression of poplar homologs in the poplar floral repressor *SVL* (*SHORT VEGETATIVE PHASE-LIKE*) delayed flowering and reduced flowering abundance in *Populus* [16]. Bulb flowers are a type of high ornamental value flower. However, it is not easy for genetic transformation to happen in the majority of bulb flowers, resulting in the mechanism of bulbous flowers being limited and only a few genes being identified and verified. For example, the genes of *TgFT1*, *TgFT2*, and *TgFT3* were isolated from tulips, and functional analysis in Arabidopsis showed that overexpression of *TgFT2* resulted in very early flowering, while delayed flowering was observed after the overexpression of *TgFT1* and *TgFT3* [17]. The overexpression of *LbrSPL9* and *LbrSPL15,* identified in lily, resulted in early flowering in *A. thaliana*, which demonstrated the regulatory function of the age pathway in lily [18]. It is recognized that bulb size and endogenous hormones are the main factors that affect the flowering in bulbs, and this has been proven in several bulb flowers, such as *Lilium* [19], *Tulipa* [20], *Narcissus* [21], and *Lycoris* [22].

The genus *Lycoris* is an important ornamental and medicinal plant that is mainly distributed in China and Japan, with a few species in Myanmar and North Korea. *L. radiata* is the typical species in the genus, with the special characteristics of curly petals and flamboyant stamens, like spiders. When they bloom, flower stems stick out of the ground and are crowned with an inflorescence containing 4–6 bright red flowers. After the flowering, the leaves begin to appear and continue growing in the fall and winter until the following spring. Due to the beautiful flowers and the long green leaf period, *L. radiata* has been widely used in garden landscapes. Commonly, the *L. radiata* flowers in early autumn; however, we also observed that the natural flowering period can vary by one month in different populations. What is the main reason causing such widely varying flowering times? We need to explore the flowering mechanism of *L. radiata*. Previous studies have focused on leaf photosynthetic performance [23], physiological and biochemical indicators such as floral bud differentiation [24], and bulb development [25]. In the research on floral bud differentiation, environmental factors and hormones were considered the main factors that affect the outcome of flower buds in *Lycoris*. Wei et al. (2019) found that temperature can affect *L. radiata* flowering; 25 °C was the optimum temperature for *L. radiata* to bloom, while blooms were inhibited at 30 °C [26]. Endogenous hormones GA3 and IAA were beneficial for the differentiation of *Lycoris* flower buds [27]. These studies provide some evidence that endogenous and exogenous factors can promote or inhibit the flowering of *Lycoris* to a certain extent but did not involve the core regulatory modules and genes, which were not sufficient to reveal the flowering mechanism. Therefore, an in-depth understanding of the molecular mechanisms regulating the flowering process is necessary to overcome the difficulties of flowering regulation in *L. radiata*.

Floral bud differentiation is a transition from vegetative growth to reproductive growth, accompanied by changes in physiology morphology. Many researchers have observed the process of floral bud differentiation by histomorphology in paraffin sections and then explored the genes related to this process by transcriptome and gene screening methods [28]. For example, the combination of transcriptomics and paraffin sectioning techniques was performed to study the differences in bud differentiation between early- and late-maturing cotton, from which *GhCAL* was identified and *GhCAL*-resistant transgenic cotton exhibited late bud differentiation and flowering time [29]. In *Hylocereus polyrhizus*, the morphological and cytological processes of floral bud differentiation at each stage were observed by paraffin sections, and then 47 core genes related to floral bud differentiation were selected by transcriptome sequencing in samples covering different stages [30]. In this study, we aim to identify the core genes related to floral bud differentiation and flowering time in *L. radiata*. Therefore, we chose three populations of early, middle, and late natural flowering time in *L. radiata* as materials. The morphogenesis of flower buds within bulbs was observed by paraffin sectioning, and the cDNA libraries of shoot apical meristems (SAMs) at different differentiation stages were constructed and sequenced by high-throughput sequencing. As a result, a large amount of differentially expressed genes (DEGs) related to the floral bud differentiation process were identified, including the genes universally involved in the process of differentiation and some involved in the genetics of different populations. In addition, nine genes were found that are not only differentially expressed in bud differentiation but also have a special expression pattern in three populations with different natural flowering times. It is hypothesized that these DEGs play a key role in regulating floral transformation. These results provide important information about the genes and pathways associated with the development of flower transformation in *L. radiata* and may help to further investigate the molecular mechanisms of flowering in *L. radiata*.

## 2. Results

### 2.1. Cytological Characteristics during Floral Bud Differentiation in L. radiata

According to the cytological changes of *L. radiata* SAMs from vegetative to reproductive growth in *L. radiata*, the whole process is divided into six stages: the vegetative growth period, the bud undifferentiated period, the leaf primordium differentiation period, the bract primordium differentiation period, the petal primordium differentiation period, and the stamen primordium differentiation period.

**Figure 1:**
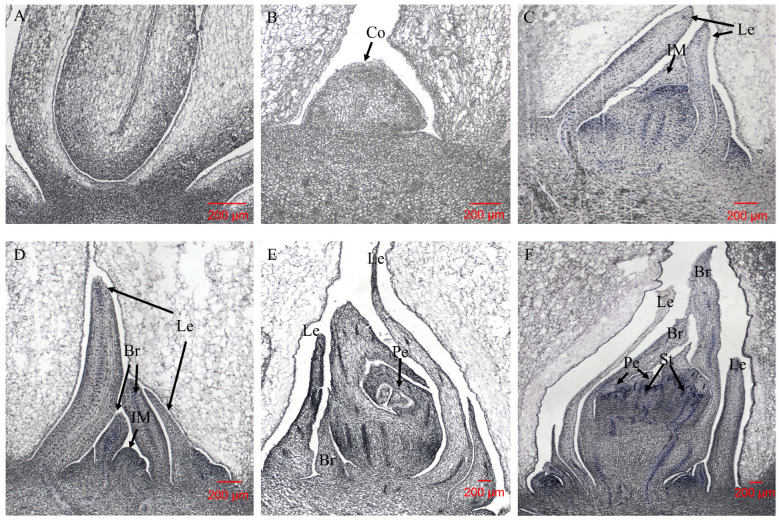
Floral bud differentiation of *Lycoris radiata* observed by paraffin slices. (**A**) Vegetative growth period; (**B**) bud undifferentiated period; (**C**) leaf primordium differentiation period; (**D**) bract primordium differentiation period; I petal primordium differentiation period; (**F**) stamen primordium differentiation period. Co = conical growing point; Le = leaf primordium; IM = inflorescence meristem; Br = bract primordium; Pe = petal primordium; St = stamen primordium. Bar = 200 μm.

In *L. radiata* SAMs, the flower buds are not seen during the vegetative growth period, when they are in a state of vegetative growth (Figure 1A). At the stage of undifferentiated flower buds, *L. radiata* flower buds are small and their apical growing points are conical, but the floral primordia are not formed (Figure 1B). In the pre-bud differentiation stage, the growing point is clearly elevated, the perianth primordia are elevated, and larger floral bud differentiation occurs, indicating that *L. radiata* has changed from nutritional to reproductive growth (Figure 1C,D). At the later stages of bud differentiation, the perianth becomes larger and the perianth primordia begin to differentiate, with the gradual appearance of stamen primordia (Figure 1E,F).

**Figure 2:**
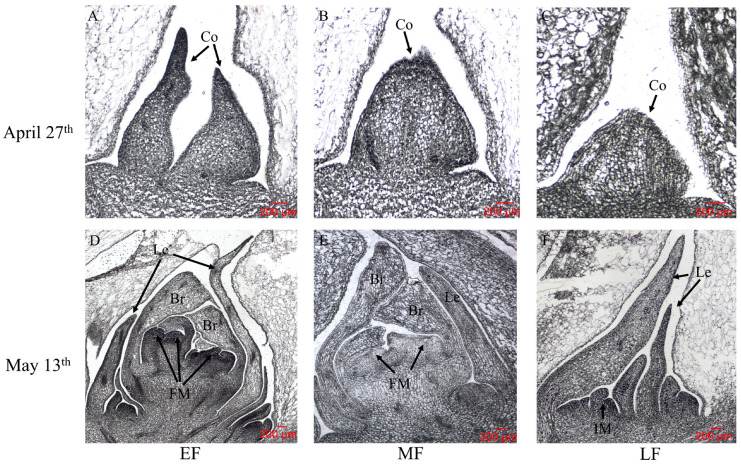
Morphological characteristics of floral bud differentiation of three populations of *Lycoris radiata;* 27 April and 13 May were the dates of collecting samples. (**A**,**D**) E1 and E2 (early flowering: EF group); (**B**,**E**) M1 and M2 (middle flowering: MF group); (**C**,**F**) L1 and L2 (late flowering: LF group). Co = conical growing point; Le = leaf primordium; Br = bract primordium; FM = floral meristem; IM = inflorescence meristem. Bar = 200 μm.

In three populations, at the same sample time, different groups showed varying development stages. They were labeled as E1 and E2 (EF group), M1 and M2 (MF group), and L1 and L2 (LF group). In the EF group, the SAM of E1 was in the pre-bud differentiation stage (Figure 2A), while the SAMs were still in the bud undifferentiated period, with an expanded conical growing point in M1 (Figure 2B) and L1 (Figure 2C). In the morphological differentiation period, the early-flowering group showed the latest stage of differentiation, with the largest and complete conical growing point (Figure 2D). In the middle-flowering group, the SAM was in late floral bud differentiation, with clear bract primordium and leaf primordium, but the conical growing point was less than in the early-flowering group (Figure 2E). Compared with the early- and middle-flowering groups, the SAM of the late-flowering group showed an early stage of floral bud differentiation, with expanded perianth primordia and clear leaf primordium (Figure 2F).

### 2.2. Statistics of Sequencing, Assembly, and Annotation of RNA-Seq in L. radiata

A total of 115.96 Gb of clean data was obtained by RNA-seq from 18 samples. After filtering out low-quality reads, splice contamination, and reads with too much unknown base N content, there was an average of 6.44 Gb of clean data per sample. More than 96.86% of the reads showed quality values ≥ Q20, and the percentage of Q30 base distribution ranged from 92.01% to 94.16%, which indicated the high quality of the sequencing data. The clean reads were assembled to 165,109 unigenes. The total length of unigenes was 226,196,677 bp, with an average length of 1369 bp. The N50 of unigenes was 1979 bp and the GC content was 41.41%, which indicate the high quality of the assembly (Table 1).

The assembled unigenes were annotated with seven functional databases, including KEGG, GO, NR, NT, SwissProt, Pfam, and KOG. As a result, 122,777 unigenes were annotated to the NR database, accounting for 74.36% of the total unigenes. In the databases of KEGG and KOG, the number of annotated unigenes was 100,063 and 99,330, accounting for 60.60% and 60.16% of the total unigenes. There were 96,085, 95,120, 94,803, and 89,517 unigenes that were annotated in the SwissProt, NT, GO, and Pfam databases, accounting for 58.19%, 57.61%, 57.42%, and 54.22%, respectively. The number of unigenes annotated by all databases was 50,188, accounting for 30.40% of the total unigenes, and 127,400 unigenes were annotated by a single database, accounting for 77.16% of the total (Table 2).

**Figure 3:**
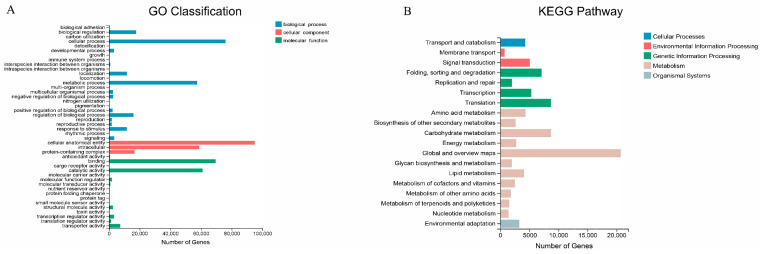
GO classification and KEGG pathways of annotated unigenes. The horizontal axis represents the number of unigenes, and the vertical axis represents the terms of GO and KEGG. (**A**) GO classification and distribution statistics; (**B**) KEGG pathways and distribution statistics.

Based on the NR annotations of the sequence homology in different species, the highest homology was found between *L. radiata* and *Asparagus officinalis* (Amaryllidaceae) (60.43%), followed by *Elaeis guineensis* (7.92%), *Phoenix dactylifera* (5.96%), *Ananas comosus* (2.19%), and *Musa acuminata *subsp. malaccensis (1.48%) (Figure S1). In GO annotation, three GO hierarchies: Biological Process, Cellular Component, and Molecular Function were classified into 44 GO terms (Figure 3A). In biological processes, the most unigenes were distributed in cellular processes, followed by metabolic processes and biological regulation. In the cellular component, the most unigenes were distributed in the cellular anatomical entity, followed by intracellular and protein-containing complexes. In molecular function, binding and catalytic activity were the most enriched terms, with a large number of unigenes (Figure 3A). In the KEGG annotation, five categories contained 19 KEGG pathways (Figure 3B). The most informative categories were Metabolism, followed closely by Genetic Information Processing and Environmental Information Processing (Figure 3B). The Metabolism category contained the most pathways and unigenes, in which the most unigenes were annotated in the Global and Overview Maps pathway (Figure 3B).

### 2.3. Differentially Expressed Genes (DEGs) Identification in L. radiata

**Figure 4:**
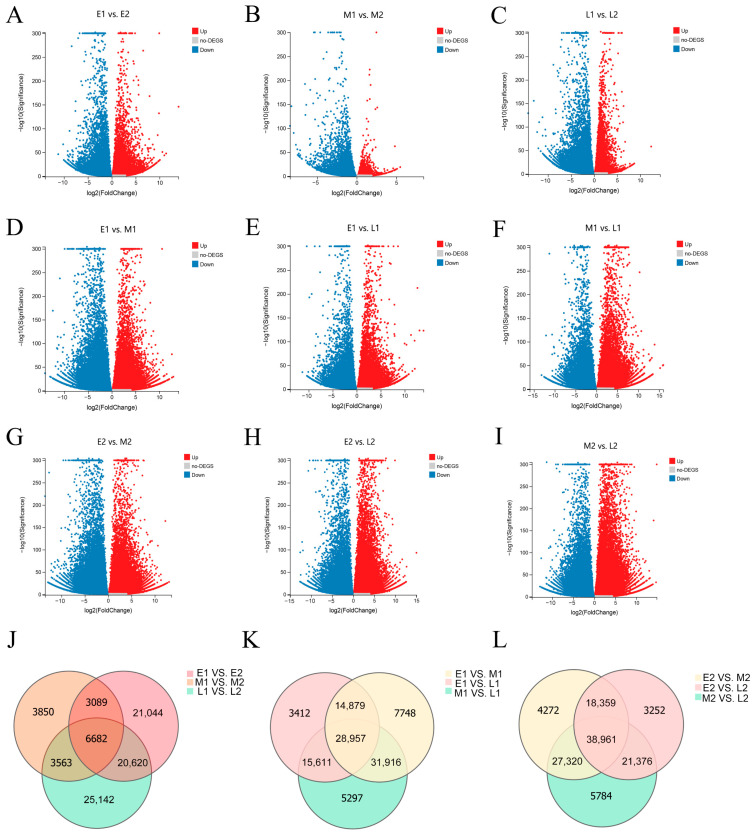
Volcano plots of DEGs (**A**–**I**) and Venn diagrams of DEGs (**J**–**L**). (**A**–**C**) Volcano plots of DEGs in the comparisons of E1 vs. E2, M1 vs. M2, and L1 vs. L2. (**D**–**F**) Volcano plots of E1 vs. M1, M1 vs. L1, and E1 vs. L1. (**G**–**I**) Volcano plots of E2 vs. M2, E2 vs. L2, and M2 vs. L2. Red dots represent up-regulated genes, blue dots represent down-regulated genes, and gray dots represent no DEGs. The x-axis represents the fold change in the difference after conversion to log2, while the y-axis represents the significance value after conversion to −log10. (**J**) Venn diagram for comparisons of M1 vs. M2, L1 vs. L2, and E1 vs. E2. (**K**) Venn diagram of E1 vs. L1, M1 vs. L1, and E1 vs. M1. (**L**) Venn diagram of E2 vs. M2, M2 vs. L2, and E2 vs. L2. Each circle represents a set of genes. The overlapping regions of different circles represent the intersection of these gene sets and are co-expressed genes. The not-overlapping parts represent the uniquely expressed genes. The numbers in the figure represent the number of DEGs in the corresponding regions.

In order to investigate the gene expression at different stages of floral bud differentiation and the key genes that regulate flowering in different populations, several comparisons were conducted between the development stages and among the three populations (Table S2). In the comparisons between the two development stages, 51,435 DEGs were detected in E1 vs. E2, including 24,709 up-regulated and 26,726 down-regulated genes (Figure 4A). Additionally, 17,184 (6361 up-regulated and 10,823 down-regulated) DEGs were identified in the comparison of M1 vs. M2, which was the fewest DEGs in all comparisons (Figure 4B). In the comparison of L1 vs. L2, 56,007 DEGs were identified, of which 22,992 were up-regulated and 33,015 were down-regulated (Figure 4C). In the same sample time, a comparison of different populations revealed that the up-regulated genes were 39,414, 43,903, and 32,219 in E1 vs. M1, M1 vs. L1, and E1 vs. L1, respectively, and the down-regulated genes were 44,086, 37,878, and 30,640 (Figure 4D–F). In the comparisons of E2 vs. M2, M2 vs. L2, and E2 vs. L2, the up-regulated genes were 42,778, 46,242, and 39,089 and the down-regulated genes were 46,134, 47,199, and 42,859 (Figure 4G–I). A comparison of Venn diagrams at different comparison times in the same population revealed 6682 DEGs that act in the common floral bud differentiation process (Figure 4J). A comparison of Venn diagrams for the same period in different populations revealed that 3412 and 3252 DEGs were specifically expressed in the comparisons of E1 vs. L1 and E2 vs. L2, 7748 and 4272 DEGs were specifically expressed in the comparisons of E1 vs. M1 and E2 vs. M2, and 5297 and 5784 DEGs were specifically expressed in the comparisons of M1 vs. L1 and M2 vs. L2 (Figure 4K–L).

To further investigate gene expression profiles, the expression levels were clustered using Mfuzz. In different development stages, twelve unique expression patterns were identified in comparisons of E1 vs. M1 vs. L1 and E2 vs. M2 vs. L2. In the early stage of development (E1 vs. M1 vs. L1), the unigenes in cluster 6 and cluster 10 showed the up-regulation pattern, but the unigenes in cluster 3 and cluster 11 presented the down-regulation pattern (Figure S2A). In the late stage of development (E2 vs. M2 vs. L2), the up-regulation clusters were 2 and 11, and the down-regulation clusters were 8 and 9 (Figure S2B).

### 2.4. GO and KEGG Annotation of DEGs

**Figure 5:**
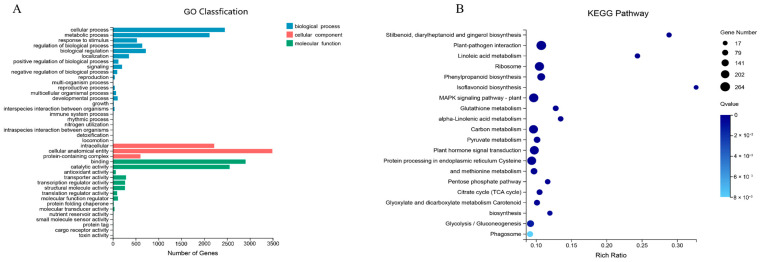
GO functional classification and KEGG enrichment analysis of DEGs identified in comparisons of E1 vs. E2, M1 vs. M2, and L1 vs. L2. (**A**) GO functional classification. The x-axis represents the number of genes, and the y-axis represents the GO terms. (**B**) KEGG enrichment analysis. The x-axis represents the enrichment rates, and the y-axis represents the annotated pathways. The size of the circle represents the number of genes, and the shade of the circle color represents the size of the Q-value.

To explore gene function, the DEGs were enriched in the GO and KEGG databases, respectively. A total of 6682 DEGs were classified in the GO function in comparisons of E1 vs. E2, M1 vs. M2, and L1 vs. L2 (Figure 4J). They were divided into three main categories of GO classification (Figure 5A). In the biological process, there are more than 2000 DEGs mainly enriched in the cellular process and the metabolic process, respectively. In the cellular component, DEGs were mainly associated with cellular and intracellular anatomical entities. For molecular function, binding and catalytic activity are the top two abundant subcategories enriched by unigenes, with more than 2500 unigenes (Figure 5A). In KEGG enrichment, a total of 6682 DEGs were enriched in comparisons of E1 vs. E2, M1 vs. M2, and L1 vs. L2 (Figure 5B). The DEGs were successfully annotated as a member in 129 pathways, and 32 KEGG pathways were significantly enriched (*p* < 0.05). Relatively more genes were annotated in the pathways of plant–pathogen interaction (ko04626; 264 genes), MAPK signaling pathway–plant (ko04016; 244 genes), ribosome (ko03010; 240 genes), plant hormone signal transduction (ko04075; 227 genes), carbon metabolism (ko01200; 225 genes), and protein processing in the endoplasmic reticulum (ko04141; 223 genes).

**Figure 6:**
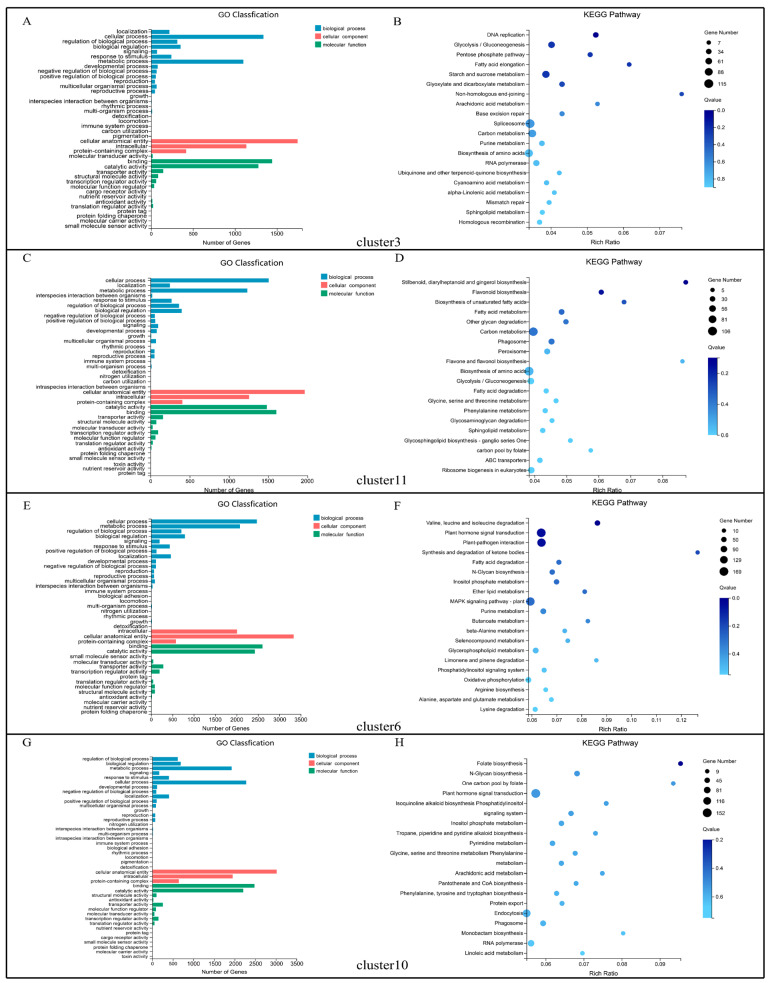
GO functional classification and KEGG enrichment analysis of DEGs in clusters 3, 11, 6, and 10 during the physiological differentiation period (E1 vs. M1 vs. L1). E, M, and L represent the early-, middle-, and late-flowering populations. (**A**,**B**) GO functional classification and KEGG enrichment analysis of DEGs in cluster 3. (**C**,**D**) GO functional classification and KEGG enrichment analysis of DEGs in cluster 11. (**E**,**F**) GO functional classification and KEGG enrichment analysis of DEGs in cluster 6. (**G**,**H**) GO functional classification and KEGG enrichment analysis of DEGs in cluster 10.

**Figure 7:**
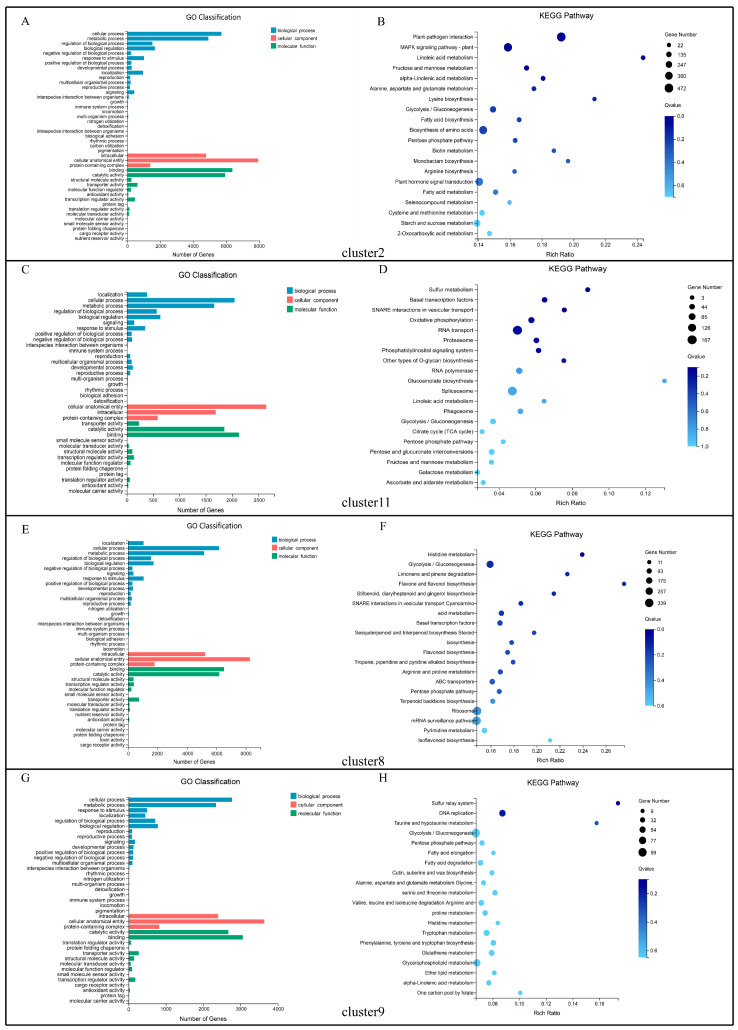
GO functional classification and KEGG enrichment analysis of DEGs in clusters 2, 11, 8, and 9 during the morphological differentiation period (E2 vs. M2 vs. L2). E, M, and L represent the early-, middle-, and late-flowering populations. (**A**,**B**) GO functional classification and KEGG enrichment analysis of DEGs in cluster 2. (**C**,**D**) GO functional classification and KEGG enrichment analysis of DEGs in cluster 11. (**E**,**F**) GO functional classification and KEGG enrichment analysis of DEGs in cluster 8. (**G**,**H**) GO functional classifica-tion and KEGG enrichment analysis of DEGs in cluster 9.

In the same sample time but with different flowering time populations, we focused on the series of genes that were continually up- or down-regulated. Thus, GO function and KEGG enrichment were also performed for the unigenes in cluster 6, cluster 10, cluster 3, and cluster 11 in the comparison of E1 vs. M1 vs. L1 (Figure 6), and cluster 2, cluster 11, cluster 8, and cluster 9 in the comparison of E2 vs. M2 vs. L2 (Figure 7). In the GO functional classification, the terms of cellular process, cellular anatomical entity, and binding contained the most unigenes in the categories of biological process, cellular component, and molecular function, respectively (Figure 6A,C,E,G and Figure 7A,C,E,G). KEGG enrichment showed that there cluster 6 and cluster 10 had the most unigenes that were enriched in the plant hormone signal transduction pathway during the physiological differentiation period (Figure 6F,H). In cluster 3 and cluster 11 (Figure 6B,D), most genes were enriched in the spliceosome and biosynthesis of the amino acids pathway. In the morphological differentiation period, the pathways of plant–pathogen interaction, RNA transport, ribosome, and glycolysis/gluconeogenesis were enriched with the most unigenes in cluster 2, cluster 11, cluster 8, and cluster 9 (Figure 7B,D,F,H).

### 2.5. Key DEGs Related to Floral Bud Differentiation in L. radiata

**Figure 8:**
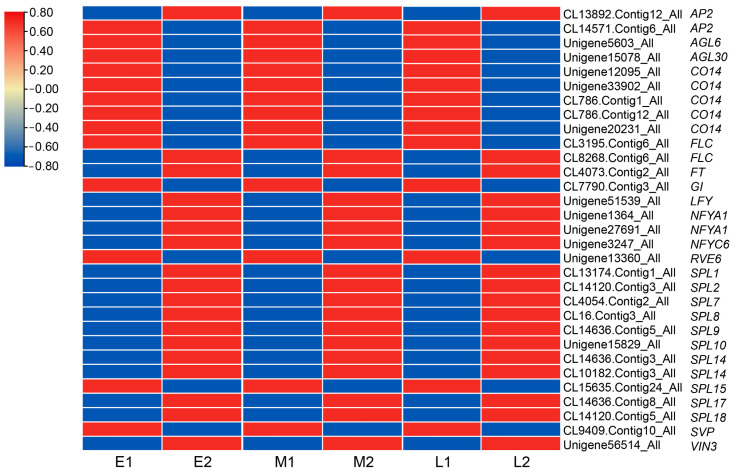
Heat map of DEGs associated with flowering time regulatory pathways in comparisons of E1 vs. E2, M1 vs. M2, and L1 vs. L2. Red means up-regulation, and blue means down-regulation.

In this study, we have identified a number of differentially expressed genes by different comparisons. In the floral bud differentiation processes (E1 vs. E2, M1 vs. M2, and L1 vs. L2), 31 unigenes that may be involved in the floral bud differentiation were sorted and the expression level shown by heatmap (Figure 8), which may regulate the flowering by different flowering pathways, for example, *AP2* (*APETALA2*), *AGL*(*AGAMOUS-LIKE*), *CO* (*CONSTANS*), *FLC* (*FLOWERING LOCUS C*), *FT* (*FLOWERING LOCUS T*), *GI* (*GIGANTEA*), *LFY* (*LEAFY*), NF-Y (Nuclear Factor Y), *RVE* (*REVEILLE*), *SPL* (*SQUAMOSA-PROMOTER BINDING PROTEIN-LIKE*), *SVP* (*SHORT VEGETATIVE PHASE*), and VIN (*VERNALIZATION INSENSITIVE*) (Figure 8). The expression levels of *CO* and *RVE* were reduced in the photoperiod pathway during bud differentiation in *L. radiata*. The expression level of *FLC* in the vernalization pathway was not uniform, either elevated or decreased. The expression level of *SVP*, which mediates ambient temperature signaling, was progressively decreased. The expression of the floral integrator gene *FT* was increasing. The expressions of the floral meristem identity gene *AP2* and *LFY* were increasing. The expression of *SPLs* in the age pathway was mostly raised. The expression levels of three genes of the NF-Y transcription factor family increased. The different expression patterns and levels of these key genes suggest that they may regulate the floral bud differentiation and flowering time by the joint action of different pathways in *L. radiata*.

**Figure 9:**
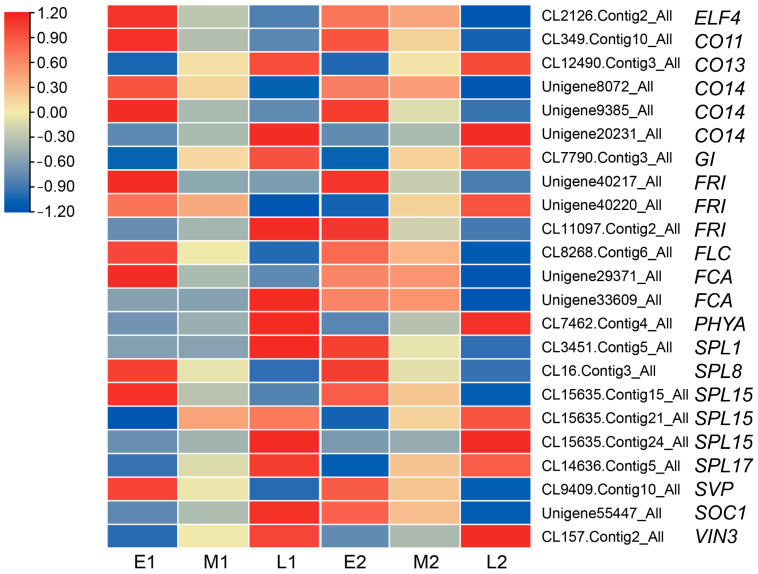
Heat map of the DEGs associated with flowering time regulatory pathways in different populations (early-, mid-, and late-flowering). Red means up-regulation, and blue means down-regulation.

Moreover, in different populations, 23 unigenes related to flowering time regulatory pathways were identified (Figure 9), such as *ELF* (*EARLY FLOWERING*), *CO*, *GI*, *FRI* (*FRIGIDA*), *FLC*, *FCA* (*FLOWERING LOCUS CA*), *PHYA* (*PHYTOCHROME A*), *SPL*, *SVP*, *SOC1* (*SUPPRESSOR OF CONSTANS 1*), and *VIN*. The genes *GI*, *PHYA*, and *VIN3* showed low expression levels in the early-flowering population and high expression in the late-flowering population. In contrast, *ELF4*, *FLC*, and *SVP* had high expression levels in the early-flowering population but low expression in the late-flowering population (Figure 9). In the physiological differentiation period, *SOC1* was expressed less in early-flowering populations compared to late-flowering populations. However, in the morphological differentiation period, *SOC1* showed an opposite expression pattern in two populations. Expression patterns of *FCA* were inconsistent across populations in the physiological differentiation period. However, it was highly expressed in early-flowering populations in morphological differentiation. *CO*, *FRI*, and *SPL* showed different expression patterns in different populations during floral bud differentiation.

In the combination of the development stages and populations of different flowering times, there were five genes that were differentially expressed not only in floral bud differentiation but also in three different populations, namely, *GI* (CL7790.Contig3_All), *FLC* (CL8268.Contig4_All), *SPL8* (CL16.Contig3_All), *SPL15* (CL15635.Contig24_All), and *SVP* (CL9409.Contig10_All). They could be candidate core genes in future studies.

### 2.6. Identification of Candidate Genes Associated with the Floral Transition by Weighted Gene Co-Expression Network Analysis (WGCNA)

**Figure 10:**
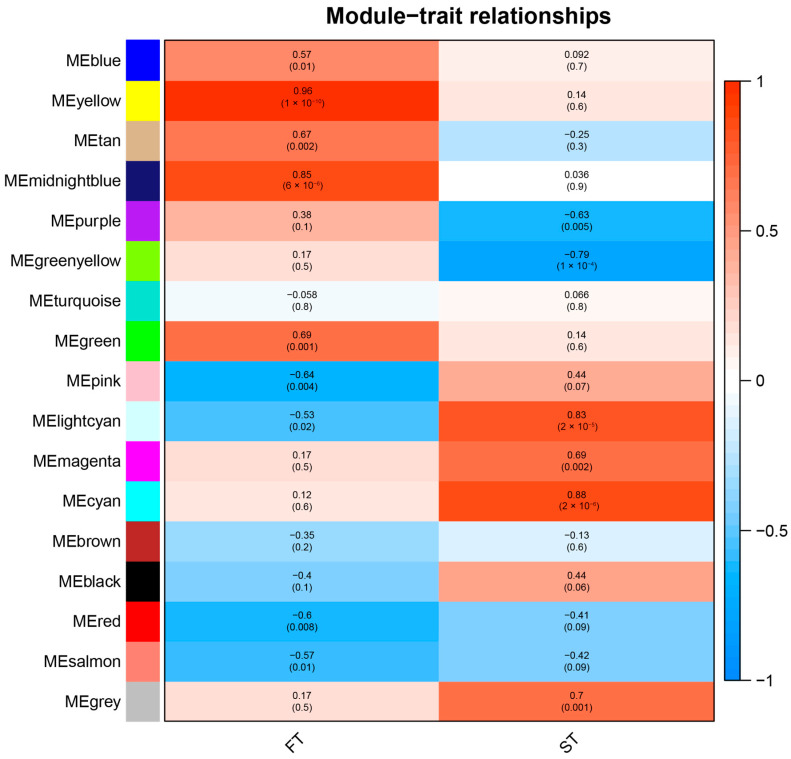
Relationship of modules and traits by WGCNA analysis. Each row corresponds to a module, and the name of the module is indicated on the left. Each column corresponds to a specific trait; FT represents the flowering time trait (comparisons of E1 vs. M1 vs. L1 and E2 vs. M2 vs. L2) and ST represents the development stages of floral bud differentiation (comparisons of E1 vs. E2, M1 vs. M2, and L1 vs. L2). The color of each cell at the row-column intersection indicates the correlation coefficient between the module and the trait. A highly positive or negative degree of correlation between a specific module and a trait is indicated by red or blue, respectively.

Weighted gene co-expression network analysis (WGCNA) was employed to further investigate the co-expression network of candidate genes. Based on the correlations and gene expression trends in all samples, co-expression networks were constructed by the R library using 38,924 genes with FPKM values greater than 2 from 18 samples. Different colors represent a specific module containing a set of highly related genes. This analysis resulted in 34 different modules (Figure 10). ST represents the sample stages of common floral bud differentiation (E1 vs. E2, M1 vs. M2, L1 vs. L2), and FT represents the early-, middle-, and late-flowering times (E1 vs. M1 vs. L1, E2 vs. M2 vs. L2). Module-trait relationship analysis showed that MEblue, MEyellow, MEtan, MEmidnightblue, and MEgreen modules were positively correlated and MEpink, MEred, and MEsalmon modules were negatively correlated in the flowering time trait. For the development stage time trait, the MElightcyan module, the MEmagenta module, the MEcyan module, and the MEgrey module were positively correlated and the MEpurple module and the MEgreenyellow module were negatively correlated (Figure 10).

**Figure 11:**
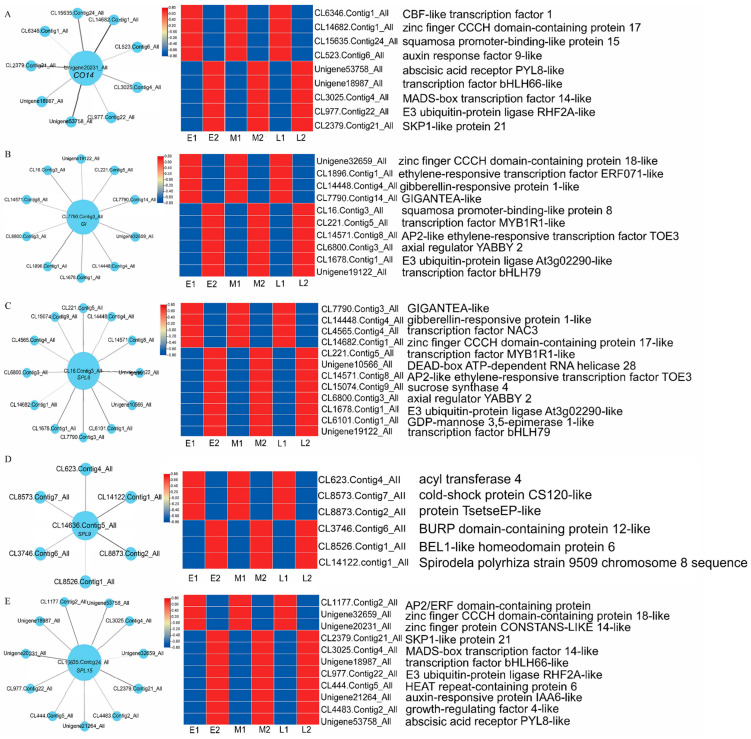
Correlation network of five hub genes related to flowering in module MEyellow. A gene network is constructed by WGCNA, in which each node represents a gene, and the connecting line (edge) between genes represents the co-expression correlation. The genes with edge weights >0.1 are visualized using Cytoscape. The size of each circle represents the number of edges. To the right of each visualized network diagram, the heatmap of correlated genes is presented to show the expression level; red and blue are up- and down-regulated, respectively. (**A**) Co-expression correlation network of *CO14* and heatmap of associated genes. (**B**) Co-expression correlation network of *GI* and heatmap of associated genes. (**C**) Co-expression correlation network of *SPL8* and heatmap of associated genes. (**D**) Co-expression correlation network of *SPL9* and heatmap of associated genes. (**E**) Co-expression correlation network of *SPL15* and heatmap of associated genes.

From these modules, five hub genes with high connectivity were found in the MEyellow module related to the flowering time trait (Figure 11), namely, *CO14* (Unigene20231_All), *GI* (CL7790.Contig3_All), *SPL8* (CL16.Contig3_All), *SPL9* (CL14636.Contig5_All), and *SPL15* (CL15635.Contig24_All). The genes linked to *CO14* (Unigene20231_All) include bHLH, CBF, MADS-box transcription factor, zinc finger CCCH domain-containing protein 17, *SPL15*, *SKP1*, auxin response factor 9-like, abscisic acid receptor PYL8-like, and E3 ubiquitin-protein ligase RHF2A-like. During floral bud differentiation, the expression of the CBF transcription factor, zinc finger CCCH domain-containing protein 17, *SPL15,* and auxin response factor 9-like was decreased, while the expression of bHLH, the MADS-box transcription factor, abscisic acid receptor PYL8-like, E3 ubiquitin-protein ligase RHF2A-like, and *SKP1* was increased (Figure 11A). The genes connected to *GI* (CL7790.Contig3_All) include MYB, bHLH, the ethylene-responsive transcription factor, *SPL8*, *GI*, *AP2*, zinc finger CCCH domain-containing protein 18-like, gibberellin-responsive protein 1-like, axial regulator YABBY2, and E3 ubiquitin-protein ligase At3g02290-like. The expression of ethylene-responsive transcription factors, zinc finger CCCH domain-containing protein 18-like, *GI,* and gibberellin-responsive protein 1-like was decreased, while the expression of MYB, bHLH transcription factors, *SPL8*, axial regulator YABBY2, E3 ubiquitin-protein ligase At3g02290-like, and *AP2* was increased (Figure 11B). The genes linked to *SPL8* (CL16.Contig3_All) included MYB, bHLH, NAC3 transcription factors, sucrose synthase4, *GI*, *AP2*, zinc finger CCCH domain-containing protein 17-like, gibberellin-responsive protein 1-like, axial regulator YABBY 2, GDP-mannose 3,5-eimerase 1-like, the DEAD-box ATP-dependent RNA helicase, and E3 ubiquitin-protein ligase At3g02290-like. The expression of NAC3 transcription factors, *GI*, gibberellin-responsive protein 1-like, and zinc finger CCCH domain-containing protein 18-like was down-regulated, but other genes were up-regulated (Figure 11C). The *SPL9* gene (CL14636.Contig5_All) was linked to acyl transferase 4, cold-shock protein CS120-like, protein TsetseEP-like, BURP domain-containing protein 12-like, BEL1-like homeodomain protein 6, and the Spirodela polyrhiza strain 9509 chromosome. With floral bud differentiation, the expression of acyl transferase 4, cold-shock protein CS120-like, and protein TsetseEP-like was decreased, while the expression of BURP domain-containing protein 12-like, BEL1-like homeodomain protein 6, and the Spirodela polyrhiza strain 9509 chromosome was increased (Figure 11D). The *SPL15* (CL15635.Contig24_All)-connected genes included MADS-box, the bHLH transcription factor, *AP2*, zinc finger CCCH domain-containing protein 18-like, *CO14*, the SKP1-like protein, E3 ubiquitin-protein ligase RHF2A-like, HEAT repeat-containing protein 6, auxin-responsive protein IAA6-like, growth-regulating factor 4-like, and abscisic acid receptor PYL8-like. During floral bud differentiation, the expressions of *AP2*, *CO14,* and zinc finger CCCH domain-containing protein 18-like were decreased, while the expressions of other genes were increased (Figure 11E).

### 2.7. qRT-PCR Validation of Expression of the Critical Flowering-Related Genes

**Figure 12:**
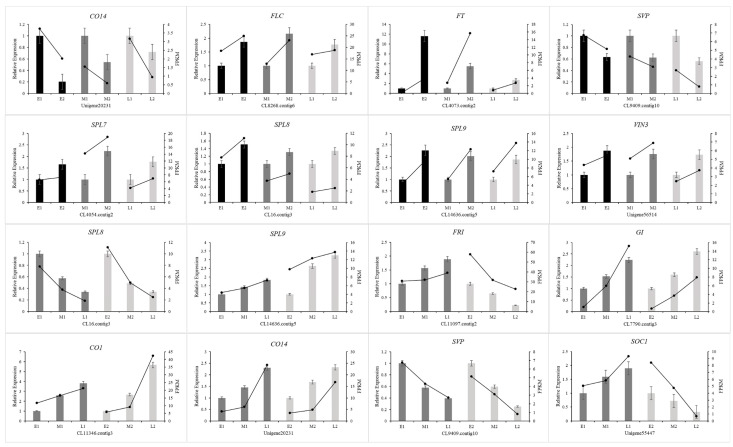
qRT-PCR analysis of genes during floral bud differentiation and in different populations. The x-axis represents different periods (upper 8 panels) and different groups (lower 8 panels). The left y-axis represents relative expression, and the right y-axis represents the FPKM value.

In this study, 12 putative candidate genes related to floral bud differentiation and flowering time were selected to validate the efficacy of RNA-seq by qRT-PCR. Eight genes (*CO14*, *FLC*, *FT*, *SVP*, *SPL7*, *SPL8*, *SPL9*, *VIN3)* were selected during the process of bud differentiation, and they were related to the regulation pathway of flowering time in different populations; four genes (*SPL8*, *SPL9*, *CO14*, *SVP)* not only played a role in the process of bud differentiation but also participated in flowering time regulation in different populations. The results show that expression patterns by qRT-PCR analysis are highly consistent with their expression trends by RNA-seq (Figure 12), indicating that the RNA-seq data is reliable in reflecting gene expression.

## 3. Discussion

Flowering is an important process of plant growth and development; it is also a significant sign of the transition from nutritional to reproductive growth [2]. The floral bud differentiation of *L. radiata* happens in spring and takes about one month. From the paraffin sections, it is presented that the meristem in the middle begins to expand first; then, the leaf primordia begin to develop, and the flower bud primordia begin to elongate and widen. The floral organs develop gradually from the outer sepals and petals to the stamens and pistils. The floral bud differentiation of *L. radiata* can be divided into six stages: the vegetative growth period, the bud undifferentiated period, the leaf primordium differentiation period, the bract primordium differentiation period, the petal primordium differentiation period, and the stamen primordium differentiation period. This is similar to the stages of floral bud differentiation observed in *Crocus sativus* [31], *Eriobotrya japonica* [32], and *Paeonia lactiflora* [33].

In this study, transcriptome sequencing was obtained from three different flowering populations at different development stages in *L. radiata*. In total, 6682 genes were differentially expressed in the process of bud differentiation; 3412 and 3252 DEGs were specifically expressed in the comparisons of E1 vs. L1 and E2 vs. L2, 7748 and 4272 DEGs were specifically expressed in the comparisons of E1 vs. M1 and E2 vs. M2, and 5297 and 5784 DEGs were specifically expressed in the comparisons of M1 vs. L1 and M2 vs. L2. This indicates that there are many genes that play a role in bud differentiation and many genes that have a significant effect on flowering time.

KEGG enrichment showed that DEGs were highly enriched in plant hormone signal transduction, suggesting that many endogenous hormones are involved in the flowering of *L. radiata* [34]. In the complex biological process of flowering regulation, hormones are a very important regulatory factor. Additionally, auxins, gibberellins, abscisic acid, and erythromycin have important effects on floral bud differentiation and flowering time [35]. It has been recognized that bulb size and endogenous hormones are the main factors that affect the flowering in bulbs, and this has been proven in several bulb flowers, such as *Lilium* [19], *Tulipa* [20], *Narcissus* [21], and *Lycoris* [22]. In the process of floral bud differentiation in *L. radiata*, *ELF* and *SVP*, two genes directly related to the gibberellin pathway [36], were found to be down-regulated during the floral bud differentiation stages in different flowering time populations. It is suggested that gibberellin may play an important role in the floral bud differentiation of *L. radiata*. Glycolysis/gluconeogenesis was also highly enriched in many DEGs, suggesting that sugar metabolism may be another important pathway for regulating flowering in *L. radiata*. In lily, the *SUT* gene, a gene involved in sucrose transport and the regulation of sugar concentration, was identified as being able to promote lily flowering by positively regulating the expression of the *FT* and *SOC1* genes [37]. Cai et al. (2020) measured the changes in soluble sugar content during the floral bud differentiation of *L. radiata* and found that soluble sugar was massively accumulated in the early stage of floral bud differentiation and largely consumed during flower organ formation [24]. This indicates that sugar concentration is important for the flowering of bulb flowers. In addition, DEGs are enriched in circadian rhythms, suggesting that the light signal may connect with hormone and sugar signals to regulate the flowering of *L. radiata* by multiple pathways.

Previous studies have shown that many transcription factors may regulate plant flowering by controlling specific gene networks such as the bHLH [38], MYB [39], BBX [40], and NF-Y [41] families. In this study, the hub genes identified by WGCNA were highly connected to bHLH, MADS-box, and MYB transcription factors (Figure 11); we speculated that they may interact with each other and have a great impact on floral bud differentiation and flowering time in *L. radiata*. Some NF-Y transcription factors were also differentially expressed during floral bud differentiation in *L. radiata* (Figure 8). In plants, NF-Ys are involved in a variety of physiological and developmental processes, such as embryo maturation, seed germination, root elongation, fruit ripening, photosynthesis, and flowering time [42]. The NF-Y complex regulates flowering time through a highly redundant and complex mechanism [43]. Multiple individual subunits of NF-Y can interact with CO and affect the transcript levels of FT and SOC1, resulting in early or delayed flowering [44]. In *L. radiata* SAMs, *NF-YA1* (Unigene1364_All, Unigene27691_All) and *NF-YC6* (Unigene3247_All) were identified and up-regulated in floral bud differentiation. In *A. thaliana*, the overexpression of *AtNF-YA1* and *AtNF-YA4* led to delayed flowering [45]. In rice, the overexpression of *OsNF-YC6* promoted flowering under LD conditions [46]. This is consistent with the changes in expression observed during floral bud differentiation in *L. radiata*. It suggests that the function of NF-Y submits varies for floral bud differentiation and flowering time among species.

The identification and clarification of core genes are of great significance for regulating flowering time at the molecular level. In this study, we also identified 17 modules of co-expressed genes of flowering time by weighted gene co-expression network analysis (Figure 10). The gene expression pattern in module MEyellow coincided with the flowering time of different populations. Five hub genes (*CO14*, *GI*, *SPL8*, *SPL9*, and *SPL15*) related to flowering time were found from module MEyellow and were confirmed to affect flowering time in *Arabidopsis*. In *Arabidopsis*, CO promotes flowering through the activation of *FT* and *SOC1* [47]. *AtGI* has been shown to promote flowering [48]. *GI* expression was reduced during floral bud differentiation and was low in the early-flowering population but high in the late-flowering population, suggesting that *GI* may negatively regulate *L. radiata* flowering. The study proved that the overexpression of *OsGI* resulted in delayed flowering times, and the effect was more pronounced under short days in rice [49]. The SPL transcription factor family plays an important role in flowering. SPL9 induces flowering by activating MADS-box genes, including *AP1*, *LFY*, *FUL,* and *SOC1* [50]. The overexpression of *SPL8* promotes flowering, whereas the down-regulation of *SPL8* moderately delays flowering [51]. *SPL15* promotes the juvenile-to-adult growth stage transition, thereby promoting the ability to respond to photoperiod induction in flowering [52]. AtSPL15 promotes flowering under SD conditions [53]. In *A. thaliana*, overexpressing *SPL10* showed precocious flowering, whereas the triple loss of the function mutants of *SPL10* and its two homologous genes, *SPL2* and *SPL11*, showed late flowering compared with wild-type plants [54]. The overexpression of *EjSPL3*, *EjSPL4*, *EjSPL5*, and *EjSPL9* all have the function of promoting flowering in *Arabidopsis* [55]. In *L. radiata*, the expression of most SPLs associated with the age pathway increased during floral bud differentiation, and only *SPL15* (CL15635.Contig24_All) decreased during floral bud differentiation and in the early-flowering population. It is hypothesized that *SPL15* negatively regulates flowering in *L. radiata,* and other SPLs may play an important positive regulation.

In addition, some genes that were involved in flowering regulation pathways in *A. thaliana* were differentially expressed in floral bud differentiation in *L. radiata*. *FLC* is a negative regulator of flowering and is involved in autonomic and vernalization pathways [56]. *FCA* promotes FT expression by repressing *FLC* in the autonomous pathway, whereas FRI inhibits flowering by promoting *FLC* expression in the vernalization pathway [57]. In *L. radiata*, the expression of *FCA*, *FLC*, and *FRI* did not follow any obvious expression pattern, which is possible since they are regulated by the crosstalk between the autonomous and vernalized pathways. These results reveal that floral bud differentiation is not the result of a single gene functional class but rather the result of the interaction of flowering pathways in *L. radiata*. However, these identified new genes provide genetic resources for the regulatory pathways of *L. radiata* floral bud differentiation and the development of *L. radiata* early- and late-flowering cultivars.

## 4. Materials and Methods

### 4.1. Plant Materials and Collection

*L. radiata* bulbs were planted in the National *Lycoris* Germplasm Bank, located in the Institute of Botany, Jiangsu Province, and the Chinese Academy of Sciences (Nanjing Botanical Garden Mem. Sun Yat-Sen). In this study, we selected three populations of *L. radiata,* with flowering times varying from mid-August, mid-September, and early October, separately. Three sample groups with different flowering times were classified as early flowering (EF), middle flowering (MF), and late flowering (LF). The floral bud differentiation of *Lycoris* generally occurs from April to May. Hence, in each group, the SAMs of the three populations were continually collected from April to mid-May. When collecting the samples, a sterilized knife was used to take a basal plate of size 1 cm × 1 cm × 1 cm and then divide it into two parts with the same label. Half of the SAMs were stored in an FAA fixative (38% formaldehyde:glacial acetic acid:70% ethanol:glycerol in the ratio of 1:1:16:1) for cytological observation. The other half portion of the SAMs was immediately frozen in liquid nitrogen and stored at −80 °C for RNA-seq and qRT-PCR experiments.

### 4.2. Cytological Observation

The SAMs of *L. radiata* stored in the FAA fixative were graduated from low to high alcohol concentrations of 30%, 50%, 70%, 80%, and 90%, respectively, over half an hour each time. Then, they were transferred to a 1/2 xylene and 1/2 anhydrous alcohol mixture transparent solution for 2 h, pure xylene transparent solution for 2 h, and then pure xylene transparent solution for 1 h. The materials were placed in a mixture of 25%, 50%, and 75% paraffin and xylene for 30 min per stage. The process was carried out in a warm oven at 56 °C; then, the materials were placed in dissolved pure wax for 30 min, and the process was repeated twice more. The paraffin blocks were trimmed and sliced with a slicer to a thickness of 10 μm [31]. Split the wax tape and separate the wax pieces by placing them in distilled water at 50 °C to spread the wax pieces. Use a clean slide to retrieve the unfolded wax sheet, adjust the position of the wax sheet so that it is in the center of the slide, mark the slide with a pencil, place the slide on the baking table to dry, and place it vertically. The slides were primely checked on a phase contrast microscope.

Then, dewaxing and dyeing were conducted on the slides. The dewaxing was as follows: xylene for 10 min, xylene for 10 min, anhydrous ethanol for 5 min, 90% ethanol for 2 min, 70% ethanol for 2 min, and distilled water for 2 min. The dyeing was performed by staining with Cole’s hematoxylin stain (Solarbio, Beijing, China) for 10 min, washing with water to remove the residual stain for 10 min, and washing with distilled water for another 5 s. The following is the dehydration: the slides were dehydrated in 95% ethanol for 2 min, then fresh 95% ethanol for another 2 min, xylene transparent solution for 5 min, and fresh xylene transparent solution for another 5 min. Finally, the slides were placed in a phase contrast microscope (Olympus BX43, Tokyo, Japan) for observation and photos; the images were further processed with Photoshop (version 19.1.9).

### 4.3. RNA Extraction, Library Construction, and Sequencing

According to the results of cytological observation, the samples with a clear differentiation period (including physiological and morphological differentiation period) were selected for RNA-seq; 0.5 g of the cryopreserved samples was taken to extract total RNA, and each sample was set up for three biological replicates. Total RNA was isolated using the Plant RNA Extraction Kit (Huayueyang, Beijing, China) according to the manufacturer’s instructions. The RNA concentration was measured using the OneDrop 1000. RNA integrity was examined using an Agilent Bioanalyzer 2100 System (Agilent Technologies, Palo Alto, CA, USA). Purified RNA for library construction and the libraries were numbered as follows: E1-r1, E1-r2, E1-r3, E2-r1, E2-r2, and E2-r3 were the libraries of physiological differentiation and morphological differentiation stages in the early-flowering population; M1-r1, M1-r2, M1-r3, M2-r1, M2-r2, and M2-r3 were the libraries of physiological differentiation and morphological differentiation stages in the mid-flowering population; L1-r1, L1-r2, L1-r3, L2-r1, L2-r2, L2-r3 were the libraries of differentiation and morphological differentiation stages in the late-flowering population.

RNA-Seq was performed by BGI (BGI Genomics, Shenzhen, China) on the DNBSEQ platform. Total RNA was processed by the mRNA enrichment method, the obtained RNA was fragmented by interrupting buffer, random N6 primers were used for reverse transcription, and then cDNA duplexes were synthesized to form double-stranded DNA. The synthetic double-stranded DNA ends were flattened and phosphorylated, and then the ligated products were amplified by PCR with specific primers. The PCR products were heat-denatured into single-stranded DNA, and a segment of bridge primer was used to loop the single-stranded DNA to obtain a single-stranded circular DNA library for sequencing. Raw reads were deposited in the NCBI database (https://www.ncbi.nlm.nih.gov/, accessed on 31 October 2022) under BioProject number PRJNA892737.

### 4.4. Assembly and Functional Annotation

After obtaining the raw reads, the reads with low quality, joint contamination, and a high content of unknown base N were filtered out to get the clean reads. The assembled unigenes were annotated by comparison with public databases, including the National Center for Biotechnology Information (NCBI) (https://www.ncbi.nlm.nih.gov/, accessed on 16 August 2021) non-redundant protein (Nr) and nucleotide (Nt) database, the Swiss-Prot protein database (https://www.uniprot.org/, accessed on 16 August 2021), the Gene Ontology (GO) database (http://geneontology.org/, accessed on 16 August 2021), the Protein Family (Pfam) database (https://pfam.xfam.org/, accessed on 16 August 2021), the Kyoto Encyclopedia of Genes and Genomes (KEGG) database (https://www.genome.jp/kegg/, accessed on 16 August 2021), the Eukaryotic Ortholog Groups (KOG) database (https://www.hsls.pitt.edu/obrc/index.php?page=URL1144075392, accessed on 16 August 2021), the Plant Resistance Gene Database (PRGdb) (http://www.prgdb.org/prgdb4/, accessed on 16 August 2021) and PlantTFDB (http://planttfdb.gao-lab.org/, accessed on 16 August 2021).

### 4.5. Gene Expression Level Analysis and Differential Expressed Genes (DEGs) Identification

DEseq2 (http://www.bioconductor.org/packages/release/bioc/html/DESeq2.html, accessed on 16 August 2021) was used to detect DEGs (differentially expressed genes), and DEGs with fold changes >2 or <−2 and adjusted *p*-values <= 0.001 were considered to be significantly differentially expressed genes. GO and KEGG enrichment analyses were performed using Phyper, a function of R. The significant levels of terms and pathways were corrected by Q-value with a rigorous threshold (Q-value < 0.05).

### 4.6. Co-Expression Network Analysis with WGCNA

An R package for weighted gene co-expression network analysis (WGCNA) was used to construct gene co-expression modules [58]. The 38,924 differentially expressed genes (DEGs) with FPKM values above 2 were identified between FT (flowering time, E1 vs. E2, L1 vs. L2, M1 vs. M2) and ST (stage time, E1 vs. L1 vs. M1, E2 vs. M2 vs. L2). The pools were analyzed by WGCNA in eighteen samples in our research. The Dynamic Tree Cut algorithm, with a coefficient of variation cut-off of 0.25 and a minimum module size of 50 genes, was used to filter out genes with low variation among the samples and to reduce hierarchal clustering. An eigengene network was constructed to represent the relationships among the modules. The networks were visualized using Cytoscape v.3.9.1 with WGCNA edge weight > 0.10 [59].

### 4.7. qRT-PCR Validation of RNA-seq Data

To validate the RNA-seq data, we randomly selected 12 genes and performed a quantitative reverse-transcription polymerase chain reaction (qRT-PCR) analysis. First-strand cDNA was generated from 0.5 μg of total RNA isolated from each of the different periods using the PrimeScriptTM RT Reagent Kit with gDNA Eraser (Perfect Real Time, TaKaRa, Dalian, China). Primers for qRT-PCR were designed using Primer Quest online software (version 2.2.3) (https://sg.idtdna.com/PrimerQuest/Home/Index, accessed on 26 August 2022) and synthesized by General Biol Co., Ltd. (Anhui, China) (Table S1). qRT-PCR was performed using a LightCycler 480 (Roche Molecular Biochemicals, Mannheim, Germany) and an SYBR Green-based PCR assay. Each 20 µL qRT-PCR reaction contained 10 µL SYBR Premix Ex TaqTMII (TaKaRa, Dalian, China), 5 µL cDNA,0.8 µL forward primer (10 μM), 0.8 µL reverse primer (10 μM), 0.4 µL ROX Reference Dye, and 3 µL dH_2_O. The qRT-PCR process was conducted as follows: 95 °C for 30 s, followed by 40 cycles of 95 °C for 5 s, and 60 °C for 31 s in 96-well optical reaction plates. The *L. radiata Actin* gene was used as an internal standard to calculate relative fold differences based on comparative cycle threshold (2^−ΔΔCt^) values.

## 5. Conclusions

In this study, we observed the cytological changes during floral bud differentiation in *L. radiata* and made a clear description of six development stages of differentiation characteristics. The key to changing from nutritional to reproductive growth happens in the pre-bud differentiation stage, followed by the physiological and morphological differentiation stages. High-throughput sequencing was performed to identify the core genes involved in floral bud differentiation and development. In three populations of different flowering times, the expression patterns of DEGs also were analyzed to further identify the core genes. These DEGs may regulate flowering time by pathways, including age, photoperiod, and vernalization in *L. radiata*. They may be the main pathways that affect flowering time by interacting with each other. WGCNA provided five hub genes (*CO14*, *GI*, *SPL8*, *SPL9*, *SPL15*) related to flowering time, which is linked to SPLs, AP2, hormone response factors (auxin, gibberellin, ethylene, and abscisic acid), and several transcription factors (MADS-box transcription factor, bHLH, MYB, and NAC3). It suggests a complex molecular mechanism of floral bud development and flowering time in *L. radiata*, which is an interactive process by endogenous hormones and the expression of hub flowering regulators. The results present a clear study from morphology and cytology to the gene regulation of flower regulation, which will facilitate the molecular breeding of flowering time regulation in *Lycoris*.

## Figures and Tables

**Table 1:** Statistics of the RNA-Seq results.

Sample	Total Raw Reads (M)	Total Clean Reads (M)	Total Clean Bases (Gb)	Clean Reads Q20 (%)	Clean Reads Q30 (%)	Clean Reads Ratio (%)
E1_r1	49.08	42.9	6.43	97.24	92.92	87.4
E1_r2	49.08	42.75	6.41	97.11	92.6	87.11
E1_r3	49.08	43.01	6.45	97.06	92.47	87.63
E2_r1	49.08	43.11	6.47	97.03	92.41	87.83
E2_r2	49.08	42.82	6.42	97.23	92.91	87.25
E2_r3	50.83	43.18	6.48	97.1	92.62	84.95
L1_r1	47.33	42.24	6.34	97	92.33	89.24
L1_r2	49.08	43.41	6.51	96.97	92.3	88.45
L1_r3	47.33	42.46	6.37	96.86	92.01	89.71
L2_r1	47.33	43.25	6.49	97.73	93.57	91.38
L2_r2	47.33	43.5	6.52	97.62	93.31	91.91
L2_r3	47.33	42.68	6.4	97.85	93.91	90.18
M1_r1	49.08	42.94	6.44	97.09	92.53	87.5
M1_r2	49.08	42.78	6.42	97	92.35	87.17
M1_r3	49.08	42.81	6.42	97.03	92.42	87.22
M2_r1	47.33	43.45	6.52	97.94	94.16	91.81
M2_r2	47.33	43	6.45	97.81	93.84	90.86
M2_r3	47.33	42.83	6.42	97.9	94.08	90.5

E1 and E2: two stages of the early-flowering (EF) group; M1 and M2: two stages of the middle-flowering (MF) group); L1 and L2: two stages of the late-flowering (LF) group. r1–r3 represent three replicates.

**Table 2:** Functional annotation of unigenes.

Database	Annotated Number of Unigenes	Percentage of Total Unigenes
Total	165,109	100%
NR	122,777	74.36%
NT	95,120	57.61%
Swissprot	96,085	58.19%
KEGG	100,063	60.60%
KOG	99,330	60.16%
Pfam	89,517	54.22%
GO	94,803	57.42%
Intersection	50,188	30.40%
Overall	127,400	77.16%

## Data Availability

Raw reads were deposited in the NCBI database (https://www.ncbi.nlm.nih.gov/, accessed on 31 October 2022) under BioProject number PRJNA892737.

## Supplementary Materials

The following supporting information can be downloaded at: https://www.mdpi.com/article/10.3390/ijms232214036/s1.
