# Supplementary material for: Identification of Differentially Expressed Genes Related to Floral Bud Differentiation and Flowering Time in Three Populations of Lycoris radiata
Source: Int J Mol Sci. 2022 Nov 14;23(22):14036. doi: 10.3390/ijms232214036 (PMC9699370; doi:10.3390/ijms232214036)
Supplement: Supplementary file 1 [file ijms-23-14036-s001.zip › ijms-2004658-Supplementary Figure.pdf]

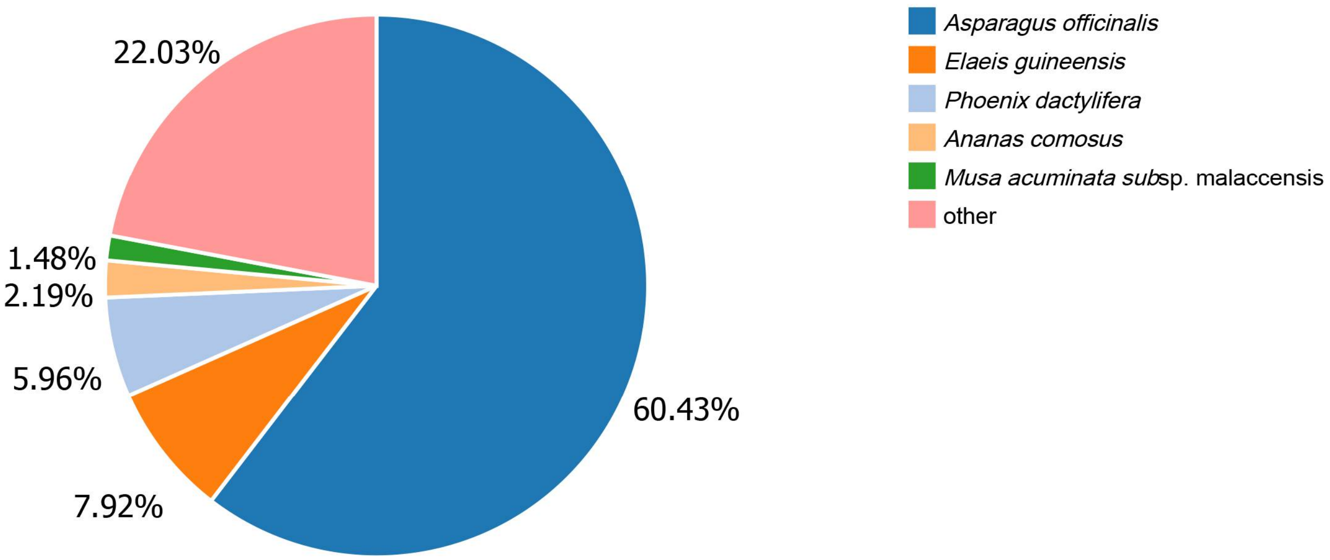

Figure S1: Distribution of Nr-annotated unigenes in different species.

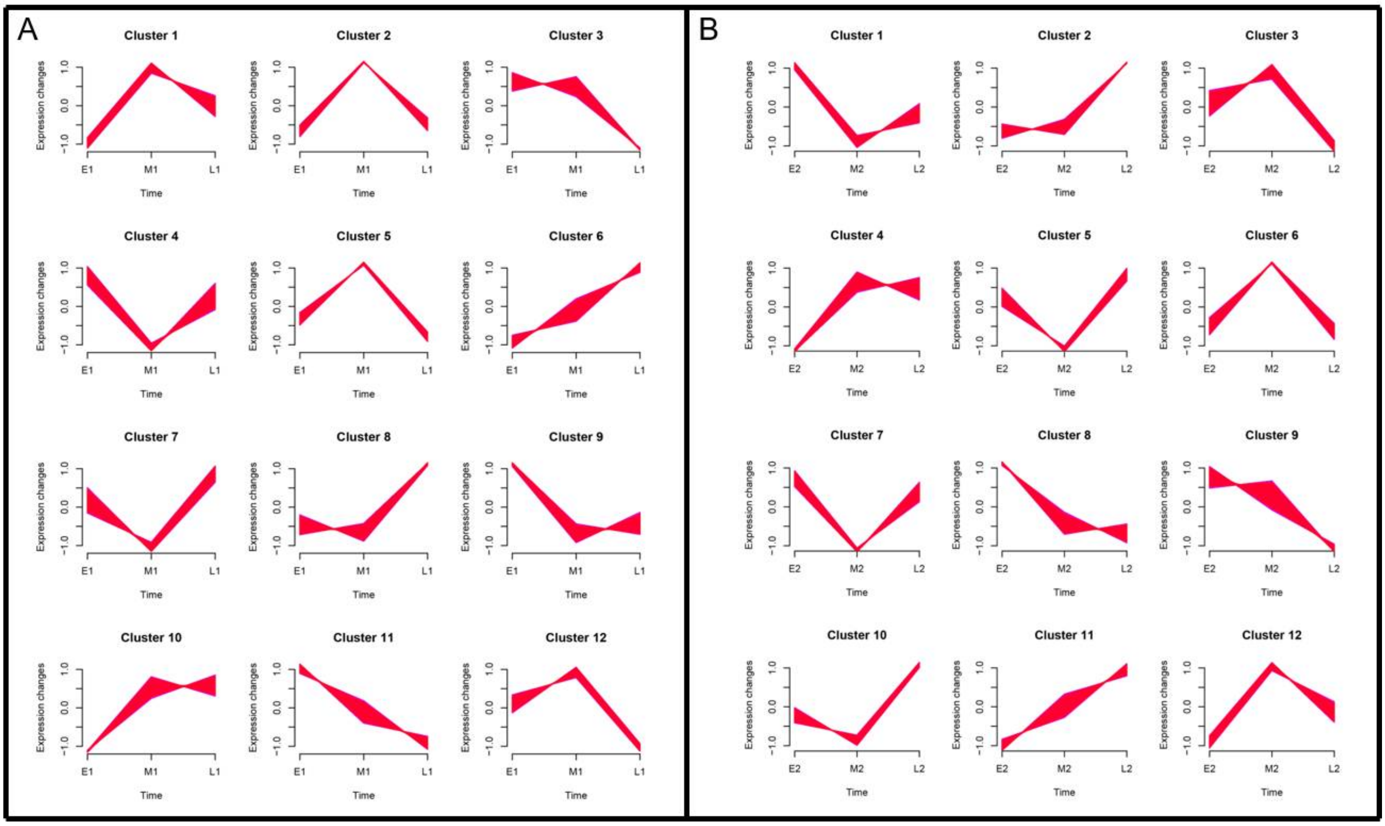

Figure S2: Time series analysis of gene expression patterns at different periods in early-, mid-, and late-flowering populations. (**A**) Gene expression patterns of the physiological differentiation period in three populations. (**B**) Gene expression patterns of the morphological differentiation period in three populations. The abbreviations of E1, M1, and L1 represent the physiological differentiation periods in populations of early flowering, mid flowering, and late flowering; E2, M2, L2 represent the morphological differentiation period in populations of early flowering, mid flowering, and late flowering.
